# Structural Segmentation of Toru Takemitsu’s Piece, Itinerant, by Advanced Level Music Graduate Students

**DOI:** 10.1177/2041669517705387

**Published:** 2017-05-05

**Authors:** Jose A. Ordoñana, Ana Laucirica

**Affiliations:** Universidad del País Vasco, Vitoria Gasteiz, Spain; Universidad Pública de Navarra, Pamplona, Navarra, Spain

**Keywords:** atonal music, music cognition, music segmentation, contemporary music, music education

## Abstract

This work attempts to study the way higher music graduate students segment a contemporary music work, Itinerant, and to understand the influence of musical feature on segmentation. It attempts to test the theory stating that saliences contribute to organising the music surface. The 42 students listened to the work several times and, in real time, they were requested to indicate the places on the score where they perceived structural boundaries. This work is characterised by its linearity, which could hinder identification of saliences and thereby, the establishment of structural boundaries. The participants show stability in the points of segmentation chosen. The results show significant coincidences among the participants in strategic places of the work, which leads us to conclude, in line with other researches, although in a work with different characteristics, that listeners can find a structural organisation in contemporary music that could allow them to understand it.

## Introduction

At the start of the 20th century, a new way to organise music emerged. A new way that was the result of a process during 19th century which broke away from the strongly hierarchic tonal structure. Imberty (2005) refers to hierarchic tonal structure as a strong organisation that should have its counterpart in the brain’s functioning. Besides, it introduces important changes regarding tones, rhythms, timbres, dynamics, registers, textures, and so forth.

An important aspect in order to understand musical works involves enculturation, a concept closely linked to the acquisition of tonal sense (Schulze, Dowling, & Tillmann, 2012; Tillmann, Bharucha, & Bigand, 2000; Wong, Roy, & Margulis, 2009). Enculturation implies two psychological factors: the aptitude to distinguish certain prototypical forms in cultural phenomena and a sufficiently high stage of development so that such recognition is possible (Imberty, 1969). Cultural environmental schemas make an important contribution to this relationship, but the apprehension of these schemas is subject or influenced by the individual’s psychic development. Thus, depending on the reference model, which has been learned mainly through the process of enculturation, Imberty (1990) formulated the hypothesis that the segmentation of a work is a true perceptual listening process which allows the listener to decode the work’s form.

Within the different stylistic traditions, some regularities can be identified with the formation of melodic, harmonic, rhythmic, and metric units. According to Krumhansl (1992), these regularities, which are internalised by the listener, affect the way he or she encodes and remembers musical sequences. They provide the basis for the interpretation of the functions of elemental events within a broader context, generating expectations for the subsequent events. This kind of schematic knowledge affects the way one encodes and remembers events. With regard to contemporary music, there exists a well-known general—albeit not total—rejection of this kind of music (Gomila, 2008; Laucirica, Almoguera, Eguilaz, & Ordoñana, 2012; Mateos-Moreno, 2014; Smith & Witt, 1989).

According to Francès (1958), conceived order does not generate perceived order. Procedures such as serialism may be coherent from the viewpoint of their conception, but the problem is whether such coherence can manifest in audition or not. This author states that, in order to understand the serial unit, the listener must acquire a different formation each time, that is, a sort of enculturation which is different each time. Meyer (1967, cited in Smith & Witt, 1989) already suggested that atonal music is difficult from a cognitive point of view because listeners are not trained in its syntax.

Lerdahl (1989a), who analyses Boulez’s “Marteau”, contemplates two types of grammar. One is called compositional grammar, which simultaneously generates piece events and serial organisation. The other is auditive grammar, employed more or less consciously by listeners, which generates the mental representations of music. Lerdahl considers compositional grammar to be inaccessible to the rest of the system, so there is a dissociation between compositional grammar and auditive grammar. In that case, there is no union between data organisation (compositional grammar) and the comprehended structure (auditive grammar). According to Lerdahl, this not only occurs in Boulez’s “Marteau” but also in many other works of so-called contemporary music. Atonal music is not stable (Lerdahl, 1989b), but it projects an event that is relatively salient from the rest. Precisely due to the lack of stability, salient events are very important from the cognitive viewpoint, and therefore, atonal music puts an end to the differentiation between salient events and structural importance. Lerdahl (1989b) states that a crucial point of his theory is the consideration of a salient contextual event in atonal music as being similar to stability in tonal music. Likewise, he admits that atonal music is not very grammatical because listeners do not have a group of logical and psychologically adequate sets to organise the surface music pitch. Therefore, the listener clings to the salient event, which is transformed into a structurally important element.

Atonal music forsakes the strongly hierarchic organisational bases of tonal music, seeking the organisation through other criteria. These criteria and their organisational bases are different from those of tonal music. Composers often use their own organisational criteria, so the listeners must intuit or discover this organisation while listening to the melodic surface, making the effort of listening and understanding the work much greater. The listeners do not feel the stability provided by the organisation implicit in tonal work, and this produces some perceptive insecurity, making their comprehension more difficult. But if the composer has undergone perceptive development in order to be able to create, listeners should be able to generate, through experience and exposure to specific contexts, the perceptive capacities that allow them to understand what the contemporary music composer writes, or at least give a logical organisation. Ordoñana and Laucirica (2010) reveal the capacity to arrange the musical surface with the first movement of “Sonate für Flöte und Klavier” by P. Hindemith that, although not included in the contemporary or nontonal repertory, can be placed within a tonally ambiguous repertory.

Rahn (1980) suggests that listeners try to organise atonal music using the skill they have acquired, that is, through tonal filters. Listening to atonal music in a tonal context submits people to cultural shock. For Imberty (2005), the generative theories in music are hampered much more by contemporary musical currents than by the music of the world because most such world music—at least, among the most well-known—is organised so that its sounds are grouped around a referent, known either as a tonic or by some other name. This could be considered as equilibrium between experience and hearing. Hence, generally, when we listen, we analyse according to tonal criteria. But when a melody does not follow the hierarchy of tonal functions, our system cannot accommodate it, and we generally cannot comprehend it (Reybrouck, 2006).

In the case of music with a nonhierarchic structure, the listener constructs a new hierarchy of rules to replace the tonal rules (Bianchi, 1985; Dibben, 1999; Imberty, 1993; Lerdahl, 1989a). Then the coherence of the musical work will depend on the listener’s capacity to treat the information outside of the conventions acquired from tonal works.

That is to say, the issue is to know how, in the absence of defined structural referents, the listener can reduce the musical surface and subsequently rebuild a system of very general temporal relations that can be applied beyond a specific case (Imberty, 1993). Thus, the theoretical principles of atonality will not be accepted or rejected only on the basis of perceptive comprehension, but, by extension, on the basis of the ease of internal hierarchic coding (Bianchi, 1985). An important aspect of hierarchic organisation is that each resulting level is an extraction of the most salient structural features of the lower levels, and hence, hierarchic organisation is based on the redundancy of information. Bianchi (1985) therefore concludes that, if atonal pitch structures are processed and retained in the listener’s memory, they could contribute to the formation of internal hierarchies.

Dibben (1994) also states that, in atonal music, as there is no tonal hierarchy, the listener must form a hierarchy of events based on other parameters. She then presents an experiment to determine whether participants deduce a hierarchy of events in atonal pieces and if so, which criterion can be used for this hierarchy. Thus, the relative salient event replaces the tension–relaxation structure of tonal music. This salient event produces a provisional hierarchy, which is always modifiable and is doubtless a feature of atonal music (Imberty, 1991). But the issue is to know how this salient event can create the equivalent of the tension–relaxation alternations found in tonal music. This means that the perceptive organisation is a hierarchy of saliences more than a functional syntactic hierarchy; that is, perceptive organisation rests on temporal phenomena more than on phenomena of functional value. Imberty (1993) concludes that the macrostructure of atonal music is made up of the hierarchisation of salient events and tensions and is the equivalent of a combination of two types of hierarchy, which Imberty calls order structure and order relation structure.

Deliège (1989) introduces a hypothesis about the existence of a mechanism of cue extraction in the construction of an outline of a musical work. Thus, it is postulated that these mechanisms would be created through attentive listening and that they could be used as labels for the retention of groups (Deliège, 1989, 1993; Deliège & El Ahmadi, 1990).

This author takes into account the role of these cues in the formation of groupings that lead to the apprehension of a global description of the work during its perception in real time. These cues allow discrimination of similarities and differences (Deliège, 1989, 1993), that is, two organisation principles that seem to underlie the auditory analysis of musical form: “the principle of sameness which constitutes groups and groupings of groups; the principle of difference which differentiates them” (Deliège & El Ahmadi, 1990, p. 20).

Once the impact of the cues is accepted, one might think that the links of similarity that are developed throughout the work are the source of the relationship between the music and the listener “and a key for the intelligibility of the general outline of the work” (Deliège & El Ahmadi, 1990, p. 21).

Deliège and El Ahmadi (1990) performed an experiment with L. Berio’s “Sequenza VI.” Their study attempted to observe the behaviour of nonmusical subjects in an unfamiliar setting in order to identify perceptive behaviours free from cultural frameworks or any other reference and to compare them with the results of musicians who are quite accustomed to this kind of music. Their work focused on the cue extraction in musical groupings. In this study, they ask: can the “listening, by which the listener tends towards maximum coincidence with the work’s structures, be used to reconstitute and analyse them?” (p. 18). The mechanisms that participate in the perception of the work’s architecture are studied in this way.

Imberty (1993) suggests “that a musical work, tonal or not, is, from the perceptual point of view, a hierarchy of changes, contrasts, perceived breaks during listening” (p. 333). Imberty (1991, 1993) presented an experiment in which he performed a perceptive analysis of Berio’s “Sequenza III” (Imberty, 1987, cited in Imberty, 1991). There are two kinds of sonorous material, which alternate with each other in this work: on one hand, some sung sequences, in which the voice ranges between certain pitches with precise intervals and, on the other hand, a series of “noise” sequences in which the voice uses all the possible forms of production. Berio alternates this sonorous material in a way that is neither regular nor always marked. For Imberty, the problem of auditive segmentation emerges in the way the listener differentiates these two types of material perceptually. This experiment involved 24 participants who did not know the work, and who had to segment it during its audition. The participants first listened to the work in order to familiarise themselves with it and, in successive hearings, they had to mark the changes, contrasts, interruptions, or any remarkable sound event.

Clarke and Krumhansl (1990) carried out three experiments using the work of K. Stockhausen, “Klavierstück IX.” Their intention was to examine three related aspects: segmentation, recall of the location, and duration of each segment in the hearers’ temporary and formal experience, but using a complete work. Dibben (1999) presented two experiments. In the first one, the goal was to investigate whether listeners hear atonal music in terms of the relative structural importance of events and whether audition is influenced by structural aspects of duration and metric. In the second one, she proposed that in the absence of clear rhythm, timbre, dynamic, and motivic information, the listener deduces relatively stable structural relationships between events in the musical surface. Addessi and Caterina (2005) compared two different types of analysis of a posttonal work, the fifth movement of the “String Quartet Op. 1” by G. Kurtág.

In the present work, we examined graduate music students’ behaviour with regard to the segmentation of a contemporary music work in real-time audition. While not forgetting the enculturation aspect, which conditions perception, poorer comprehension of nontonal music compared with tonal music may derive from the supporting structure of nontonal music and its relationship with human perception and cognition. The composer implements all the psychological resources derived from his human condition to create a work. The listener’s intuitive-perceptive capacity should allow him to organise the musical surface in order to make it comprehensible. As noted by Baroni (2010), it is not the listener’s goal to discover the composer’s composing strategies but to understand his results and interpret his aesthetic intentions. Deliège and El Ahmadi (1990) ask, with regard to aural analysis, if “at the end of listening, the listener will have understood, without other recourse, what particular type of form is concerned” (p. 18). Thus, from a perceptive viewpoint, we think the listener will be able to organise the musical surface of a work such as “Itinerant” by Toru Takemitsu.

## Method

We analysed the work from a perceptive perspective. The participants were students of advanced music studies. The experiment was carried out in two sessions separated by a four-week interval. The students did not know the work they had to listen to, and this was an essential requirement. Moreover, they were specifically given instructions to avoid contact with this work in between sessions. The participants listened to the work three times (twice in the first session and once other in the second session). They had to mark the segmentation cuts on the music score as they perceived them in real time. The scores were given at the start of the audition of the work, which was listened from start to finish without disruption. They were collected after finishing the audition. Next, we performed an analysis of the interpretation of students.

### Participants

Participants were 42 students who were studying at higher conservatories. Their age ranged from 19 to 46 years (*M* = 23.6, *SD* = 4.6). They all studied music for more than 10 years. A total of 26 participants played string instrument, 5 played keyboard instruments, 10 played wind instruments (among them one, flute), and 1 studied analysis. Regarding the election of students, as it is a perceptive, nonanalytical test, their musical experience was relevant, but not their academic specialisation. They all had some experience in the interpretation of contemporary music works. They were grouped according to their timetables within the academic organisation. The participants had to take part in both the proposed sessions.

### Instruments

The target work of the study was “Itinerant” by T. Takemitsu, with a duration of 4′20″. The version employed for the audition was that of Robert Aitken in a recording of Naxos (DDD 8.555859) produced by Silver and Kraft (2003). This work was selected due to certain characteristics previously established for this investigation. We sought a contemporary music work written for a single instrument. This characteristic was considered because works with more complex textures would hinder the analysis of the musical surface. In this sense, we followed the characteristics of the works chosen, for example, by Deliège (1989), Deliège and El Ahmadi (1990), and Imberty (1991, 1993). This work uses a traditional score. The chosen works also had to be unknown to the participants, and this was very important. Taking into account that these students were from the highest stage of musical formation, this was not easy because their musical repertory was extensive. Moreover, the works should have some kind of sonorous register.

The score of Takemitsu presents a continuous, permanently varied discourse, which is expressed with the same intervallic and dynamic material. This continuous discourse displays a linear movement in which each group could be a variation of the same idea, thereby continuously developing until the end of the piece. The tests were carried out in the participants’ conservatories on the provided audio players.

### Procedure

In the first session, the participants listened to the proposed work, “Itinerant,” by T. Takemitsu, two times, one right after the other. In the first audition, the students listened to the work in order to become familiar with it (Deliège & El Ahmadi, 1990; Imberty, 1993; Krumhansl, 1996). In this first audition, the participants did not know what they had to do, and they carried out this audition without the score. In the second audition, they were given the score of the piece and requested to mark the cuts or segmentations they perceived with a vertical line across the staff while listening in real time. They were not allowed to look at the score until the music started to sound. They should be guided by their perceptive intuition, derived from their experience as listeners and interpreters. The decision we took of requesting them to reflect the segmentations in the music score allows for a group evaluation with a simple and attainable medium. As a previous reading is not permitted and the segmentation is performed in real time, the participants—people with great audition and musical interpretation experience—are aware of the importance of sound in music, that is, what is being constructed from what is written in the music score, although it sometimes may contradict the musical score. The second session was to confirm their response; participants listened to the proposed work and were requested to perform the same task once again. The participants did not know either what they had to listen to in this second session or what they had to do. The segmentation points marked in this session were compared with those marked in the first session, so we could assess the participants’ confirmation or response stability.

## Results

### Cuts Performed by the Participants. First and Second Sessions

Participants were requested to perform the same task in both sessions, except that in the second session, there was no prior audition to become familiar with the work. There were some segmentation points in the second session that were absent in the first session and vice versa (points in the first session that were absent in the second one). The total number of points of both sessions was counted, taking into account this aspect. Thus, a total of 46 points were counted.

**Figure 1.**
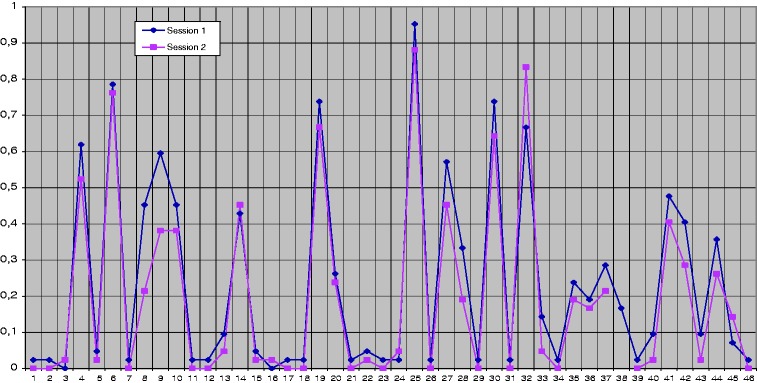
Correspondence quotients of the participants’ responses in the two sessions.

The analysis of these data allowed us to determine the possible correspondence between the responses of the two sessions. For this purpose, we calculated the correspondence quotient (Deliège & El Ahmadi, 1990) by dividing the number of participants who segmented in each session by the total number of participants (42). Comparison of the responses of the two sessions showed that the participants’ response tendency was similar or identical in both sessions. Figure 1 shows the 46 segmentation points (*x* axis) and the correspondence quotients of each point in each session (*y* axis). We also verified that the most salient points in the participants’ responses were 6, 19, 25, 30, and 32.

We then selected the most salient points at which more than 50% of the participants had segmented. These points are 4, 6, 9, 19, 25, 27, 30, and 32. We calculated Pearson’s correlation to assess the correspondence between the participants’ responses in the first and second session. These data provide a highly significant correlation (*r* = .970), which indicates that the participants’ responses are reliable and the selected segmentations have perceptive-cognitive stability. The analysis of the points at which more than 50% of the participants segmented revealed a significant correlation between the two sessions (*r* = .806).

### Number of Different Participants Who Segmented at Each Point

Although we found some points appearing in one session but not in the other, we consider this unimportant because few participants chose these segmentation points (1, 2, or 3 participants). However, most of the points chosen in the first session were also chosen in the second session, but not necessarily by the same participants. To analyse the participants’ degree of response stability, we determined how many participants segmented at a certain point, taking both sessions into account.

### Degree of Stability of the Segmentation Points

We analysed the degree of stability of each point as a function of the participants’ responses. For this purpose, we considered the number of different participants who segmented at a certain point in both the first and the second sessions. We calculated the stability quotient of the points by dividing the number of different participants who confirmed the segmentation at a certain point (participants who having segmented at a point in the first session segmented also at the same point in the second session) by the total number of different participants who cut at that point. Table 1 shows the segmentation points, the number of different participants who segmented at each point, the number of participants who confirmed their response at the same point, and the stability quotient. The mean stability quotient of the segmentation points was .419, with a maximum score of 1. Table 1 shows that, at some points, the stability quotient was 0. This is because none of the participants confirmed their response at this point. In any event, except for points 22 and 43, points with a quotient of 0 (17 points) only have one cut.

**Table 1.** Cut-Point, Number of Different Participants, Number of Participants Who Confirmed, and Stability Quotient.

Cut-point	Number of different participants	Number of participant repetitions	Stability quotient of cut-points	Cut-point	Number of different participants	Number of participant repetitions	Stability quotient of cut-points
1	1	0	0	24	2	1	.5
2	1	0	0	25	42	35	.833
3	1	0	0	26	1	0	0
4	30	18	.6	27	28	15	.535
5	2	1	.5	28	17	5	.294
6	38	27	.71	29	1	0	0
7	1	0	0	30	33	25	.757
8	24	4	.166	31	1	0	0
9	29	12	.413	32	39	24	.615
10	25	10	.4	33	6	2	.333
11	1	0	0	34	1	0	0
12	1	0	0	35	14	4	.285
13	5	1	.2	36	13	2	.153
14	26	11	.423	37	14	7	.5
15	2	1	.5	38	13	4	.307
16	1	0	0	39	1	0	0
17	1	0	0	40	4	1	.25
18	1	0	0	41	24	13	.541
19	34	25	.735	42	25	4	.16
20	17	4	.235	43	5	0	0
21	1	0	0	44	21	5	.238
22	3	0	0	45	8	1	.125
23	1	0	0	46	1	0	0

As with the correspondences, here too, we indicate the points at which more than 50% of the participants segmented. These points are 4, 6, 8, 9, 10, 14, 19, 25, 27, 30, 32, 41, 42, and 44 in Table 1. It is interesting to observe that, despite being the points with more cuts, some of these points show low stability, as in the case of point 8 (.166), point 42 (.16), or point 44 (.238). The mean stability quotient of the points at which more than 50% of the participants segmented was .508, which is higher than the mean stability quotient of all the points (.419). However, in spite of this value, it should be taken into account that we found points with high-stability quotients, in contrast to those mentioned above (8, 42, and 44). For example, the stability quotient of points 4, 6, 19, 25, 30, and 32 was higher than .6.

Thus, the respective stability quotient means that emerged from the analysis can be summarised as follows:

Mean taking into account all the points (46): .419

Mean taking into account only the segmentation points with more than 50% of cuts: .508

Table 2 shows the points with more segmentations, the points with more stability (the highest stability quotients), and the number of cuts performed by the participants. We may notice that the points of segmentation are the same. The number in parenthesis refers to the number of confirmations that appear at that point.

**Table 2.** Points Arranged From Highest to Lowest in Number of Cuts and Stability Quotients.

According to the number of cuts			According to the stability quotient		
Points	Number of cuts	Stability quotients	Points	Stability quotients	Number of cuts
25	42 (35)	.833	25	.833	42 (35)
32	39 (24)	.615	30	.757	33 (25)
6	38 (27)	.71	19	.735	34 (25)
19	34 (25)	.735	6	.71	38 (27)
30	33 (25)	.757	32	.615	39 (24)
4	30 (18)	.6	4	.6	30 (18)

In spite of the above comments about the low stability of some points (8, 42, 44), in Table 2, it can be observed that the six points at which more participants cut (25, 32, 6, 19, 30, and 4) are precisely the most stable points, although in a different order, with the notable exception of point 25, which has the highest number of cuts and is also the most stable point. This shows that the more cuts received by a segmentation point, the more stable it is.

Next, we calculated Pearson correlation between the quotient of number of cuts at a point (the number of different participants who cut at a certain point divided by the total number of participants, 42) and the stability values of that point (see Table 1). The correlation was significant (*r* = .825).

### Degree of Participants’ Stability

In this case, we analysed the stability of the participants’ responses. For this purpose, we assessed the responses of all 42 participants. We counted the number of different cuts or segmentations performed by the participants out of the 46 possible cuts. We then counted the number of segmentations confirmed in the second session. Finally, we divided the number of confirmations by the total number of cuts performed. Thus, we extracted the participants’ stability quotients.

The participants’ mean stability quotient was .484. As with the stability of the points, if only the points at which more than 50% of the participants’ cuts are considered, the mean stability quotient rises to .552. This increase is predictable because the chosen points were those that had more cuts and, as already observed, they were also the most stable.

Thus, the respective stability quotient means that emerged from the analysis can be summarised as follows:

Mean taking into account all the points (46): .484.

Mean taking into account only the cut-points with more than 50% of cuts: .552.

**Figure 2.**
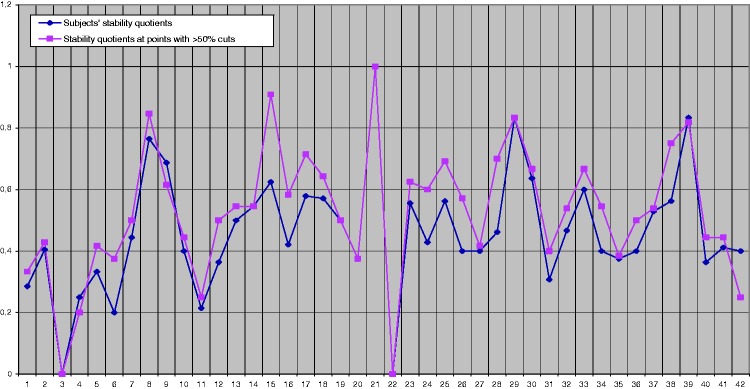
Comparison of participants’ stability quotients.

Figure 2 presents the difference in the participants’ stability when assessing all the points or the points with more than 50% of the cuts. It can be seen that most of the participants (*n* = 31, 73.8%) showed a notable improvement in their response stability at the most relevant points taking into account the number of cuts. Exceptions to this tendency were seen in participants 3 and 22, who displayed a stability quotient of 0; participants 4, 9, 39, and 42, who showed less stability when considering the points at which more than 50% of the participants cut; and participants 14, 19, 20, 21, and 29, who showed the same stability. Participant 21, with a maximum stability of 1 in both sessions, was notable.

**Figure 3.**
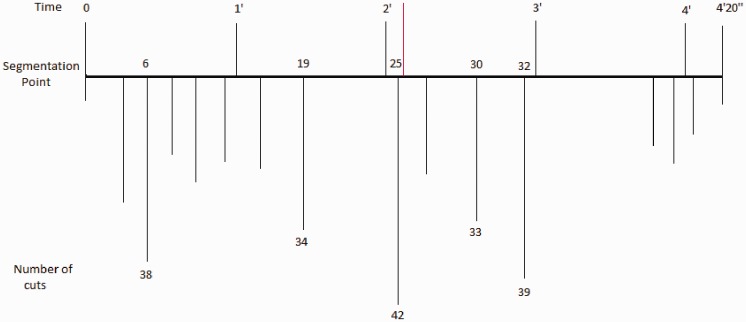
The time in minutes appears at the top, the second line corresponds to the segmentation points in time, and the numbers at the bottom refer to the number of cuts at these points. The red line corresponds to the mid-point of the work (2'10”).

The Pearson correlation between the stability quotients of all the points and the stability quotients of the points receiving more than 50% of the participants’ cuts was significant (*r* = .919). The stability quotients had values ranging between 0 and 1.

### Brief Extension of the Musicological Analysis

The “Itinerant” of Takemitsu is a work without clear sections delimiting the musical surface, except for the groupings suggested by the occurrence of silences. It is a continuous and linear piece. However, the results of the tests carried out with the participants show that perceptive intuition leads to segmenting at certain places of the musical surface more than at others. These are probably the most notable points of the piece, as noted by Deliège (1989) in effect, as of a certain degree of complexity, perception cannot capture in real time all the information received, and this is true for the auditive modality and for other sensory modalities.

After the points with a greater number of segmentations and the most stable points were established, we verified on the music score the moments of the musical discourse to which these points corresponded. From an intuitive-perceptive perspective, we observe segmentations that could somehow articulate the musical surface and discourse. This work of Takemitsu is expressed on a score without bars, so we measure the time points to identify a specific segmentation point of the score. The interpretation used in this work has a total duration of 4′20″. Table 3 shows the relation between the moments of the musical discourse at which there are segmentations, the cut-point corresponding to that moment, the number of cuts of each point, and the stability quotients. For this purpose, we considered the values of the points with the highest number of cuts, as shown in Table 2, and we arranged them from highest to lowest number of segmentations.

**Table 3.** Situation on the Score (in Minutes and Seconds) of the Points With the Most Cuts and Higher Stability.

Time	Point	Number of cuts	Stability quotient
2′09″	25	42	.833
2′56″	32	39	.615
0′29″	6	38	.71
1′31″	19	34	.735
2′38″	30	33	.757

Figure 3 presents a diagram of the course of the musical discourse with the most relevant points as a function of the participants’ response. The red line corresponds to one half of the work (2'10”). Table 3 and Figure 3 show that the most relevant point of the score—25, according to the participants’ responses—is right in the middle of the work, as though they wished to split it into two halves. Likewise, it can be seen that two more points—19 and 32—are almost symmetrical with regard to the central point (25).

**Figure 4.**
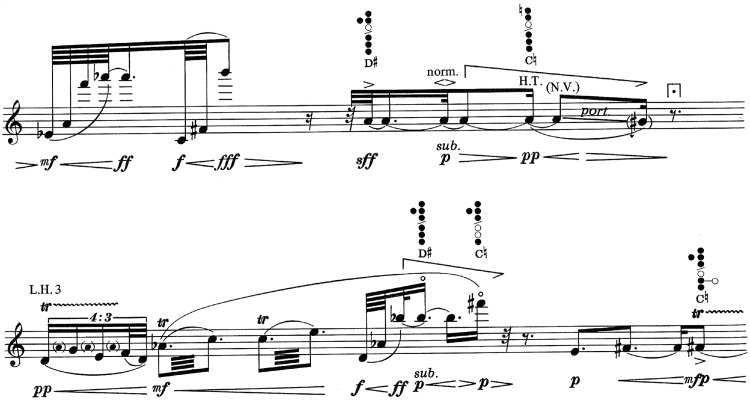
Point 25. This point is placed in the end of the first staff. Source: ©1990 Schott Music Co. Ltd., Toky.

Imberty (1993) noted that it is broadly accepted that the mind organises perceived events hierarchically. However, as noted by Lerdahl (1989b), Deliège (1989), Deliège and El Ahmadi (1990), and Imberty (1991, 1993), in contemporary music, as there are no strongly hierarchic structural points like in tonal music, organisation of the surface is supported by other elements that are nonfunctional (intensity, pitch, timbre, dynamic, etc.), but they seem to stand out on the surface of the musical discourse. It would be necessary to analyse what occurs at these points, which somehow seem to articulate the discourse of this piece of Takemitsu.

Robinson (2011) notes that “Itinerant is unmetered, constructed with a series of short, gesture-based motives separated by varying degrees of rest” (p. 68). This author divides Itinerant into six sections. The first section ends at time 1'31'' (in the interpretation used in this research) and corresponds with point 19 of the segmentation of our study (see Table 3 and Figure 3). The second section, according to the analysis of Robinson, begins at this point to finish at time 2'09'' which corresponds to point 25 of the segmentation of our study, the central point of the work and the most significant, according to the participants’ segmentations. Robinson (2011) notes this point as the beginning of the third section, adding “the third and fourth sections can be combined into a transitional passage to the fifth and sixth sections” (p. 73). This fifth section would begin at time 2'38'' which corresponds to segmentation point number 30 (see Table 3 or Figure 3), which would end at time 3'43'' which is where the sixth section starts, which corresponds to segmentation point number 41 of our study. This author particularly mentions the “whistle tones” that “appear just before the final climax of the work, creating the most drastic dynamic contrast in the piece” (Robinson, 2011, p. 77). This point corresponds to segmentation point number 32 of our study (see Table 3 and Figure 3).

After a fairly linear beginning that uses the same material, evolving and developing, gaining tension little by little, based especially on dynamic elements, in the middle of the work (2'09”), at point 25 (Figure 4), some multiphonics appear that had been previously announced, interrupting the linear character. In addition, the intensity increases to forte and fortissimo, to relax with a pianissimo, a “portamento” and without vibrato, the place where the passage that comprises point 25 ends. From here on, a trilling motive begins, which may seem more like a “tremolo,” which rapidly increases from pianissimo to fortissimo.

**Figure 5.**
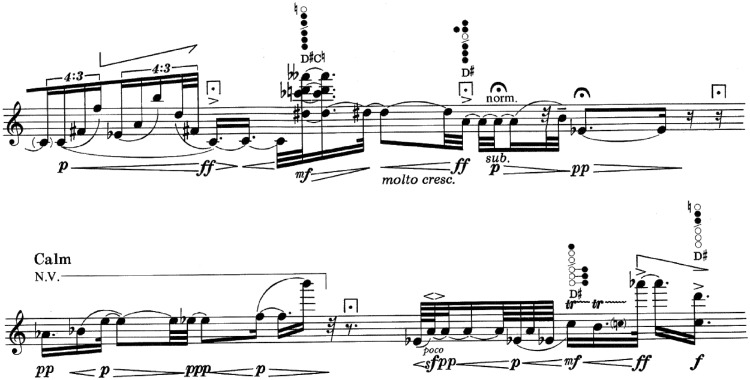
Point 19. This point is placed in the end of the first staff. Source: ©1990 Schott Music Co. Ltd., Toky.

At point 19 (1′31″; Figure 5), we find a passage similar to the former one where some multiphonics stand out in the musical discourse to subsequently relax towards a pianissimo at point 19. From here on, a melodic and calm motive begins.

**Figure 6.**
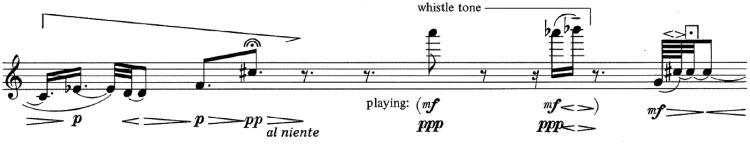
Point 32. This point is placed in the middle of the staff. Source: ©1990 Schott Music Co. Ltd., Toky.

At point 32 (2′56″; Figure 6), we find a new element that only appears at this point in the score: “Whistle tones” interpreted in a pianissimo that confer a different character to the musical discourse.

**Figure 7.**
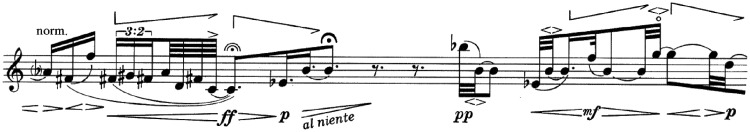
Point 6. This point is placed in the middle of the staff. Source: ©1990 Schott Music Co. Ltd., Toky.

At point 6 (0′29″; Figure 7), we may find an accelerando indicated expressly by the author, also reinforced by the written figuration. At the same spot, a crescendo to fortissimo appears where it lengthens with a very long “fermata.” From here, it evolves with a sudden diminuendo to piano to continue with an indication “al niente” with a long “fermata” over the B note. Here, the segmentation point 6 is located. It continues with a diminished octave jump (Bb-B) pianissimo followed by an accelerando.

**Figure 8.**
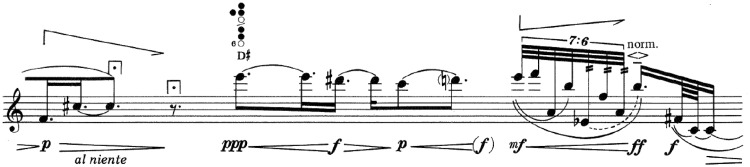
Point 30. This point is placed in the rest after C sharp. Source: ©1990 Schott Music Co. Ltd., Toky.

At point 30 (2′38″; Figure 8), we may find a very similar situation to point 6; it is the same figuration but two semitones higher. The dynamic indications are the same. The “fermatas” are different; at point 6, they are very long and long, and at point 30, they are shorts. At the segmentation point, we may find two-dotted quaver rests at point 6 and a dotted quaver with a short “fermata” at point 30. At this segmentation point, the continuation is different than that of point 6, more specifically referring to figuration.

## Discussion

With this study, we can confirm the methodology used in the works carried out by Deliège (1989) and Deliège and El Ahmadi (1990), that is, the establishment of segmentation cuts. However, the difference with regard to the above-mentioned works is that, in our study, we worked with students of higher institutions of music studies, whereas Deliège and Deliège and El Ahmadi worked with musicians and nonmusicians. In our case, the segmentations were performed directly on the score because the participants were able to follow it in real time. This allowed them to place the cuts precisely.

Likewise, we confirmed the decisive influence of the most salient elements of the piece to form the groups, which coincides with the works of Imberty (1987, cited in Imberty, 1991). These salient elements produce the strongest segmentations. Clarke and Krumhansl (1990) asked the participants to assess, on a scale ranging from 1 to 7 (1 for a soft limit and 7 for a strong limit), the greater or lesser strength of the limit or segmentation marked. In our work, this assessment of the strength or weakness of the limit or segmentation is measured by the frequency, that is, the number of cuts at a point. We consider that the more participants who cut at a certain point, the stronger will that point be from a perceptual point of view. A large part of the segmentations are performed at places where there is silence. However, we can conclude, like Deliège and El Ahmadi (1990), that silence by itself is not definite in the construction of groups because the concourse of some kind of salient event is necessary to form an important segmentation (depending on the number of cut-points). In effect, although many cuts are made during silences, not all the points have the same number of cuts, so some element other than silence must influence segmenting. This is also coherent with the theory (Clarke & Krumhansl, 1990; Deliège, 1987, 1989; Deliège & El Ahmadi, 1990; Imberty, 1991, 1993; Lerdahl, 1989a, 1989b) indicating that, in the absence of a hierarchy of pitch as in tonal music, hierarchy in atonal music is based on the most salient events of the music score. This confirms Imberty’s (1993) statement that, in atonal music, the listener constructs new hierarchic rules that replace the tonal rules, and that the coherence of the musical work depends on the listener’s capacity to process the musical information outside of the conventionalisms acquired through audition of tonal music.

An important contribution of this work is the testing of the hypothesis of Deliège (1989), in which she notes that the mechanisms to extract cues in perceptive analysis involve the formation of patterns as a function of their repetition. Also, Bianchi (1985) notes that an important aspect of hierarchic organisation is that each resulting level is an extraction of the most salient structural features of the lower levels, and therefore, hierarchic organisation is based on redundancy of information. That is, they consider repetition or redundancy as an important indicator of salient events. In the case of Takemitsu, we refer to a work in which the developments emerge from previously used material, variations of the same idea that develops continuously; that is, a work that does not use recursion to advance the musical discourse. In any event, we can recognise the use of continuous development as the “redundancy of information” of Bianchi. However, as we can deduce from the results of the work with Takemitsu, in spite of the work’s linearity, perceptive intuition organises or replaces tonal hierarchy, with another hierarchy that allows one to organise the salient elements and that facilitates comprehension of the work, as noted by Imberty (1993). The absence of these salient elements could create a waiting or “tension” in the listener, leading him to seek, from a perceptive viewpoint, that salient moment of the musical surface that will allow him to articulate and organise the discourse. As noted by Deliège and El Ahmadi (1990), the absence of the index arouses attention to the detection of a change, a crucial moment in the organisation of the form. This may be the basis for cut number 25 of the work of Takemitsu, at which all 42 participants marked a segmentation coinciding with the mid-point of the piece and at which we find the events we described in the results.

Bianchi (1985) proposes that, from an experimental work, we will know whether the pitch structures in atonality are processed by the listener or whether they are only architectural constructions that facilitate composition. The stability shown by the segmentation points and by the participants could confirm the statements of Bianchi (1985), indicating that atonality is not accepted or rejected only on the basis of perceptive comprehension, but rather, by extension, also based on the ease of internal hierarchic coding. We can then deduce that this stability could somehow emerge from the hierarchic coding capacity of the salient elements of the musical discourse. As mentioned, according to the results of this study, the most stable points are precisely those that coincide with the most salient moments. Lerdahl (1989b) notes that, due to the absence of stable conditions (those produced in tonal music), the salient event is very important from a cognitive viewpoint, and Imberty (1991) proposes that the main issue is to determine how this salient event can create the equivalent of tension–relaxation alternances found in tonal music. Some points are more stable because other points are more unstable. Perhaps, the alternation alluded to by Imberty could be explained by the movement created between two more stable points that coincide with the most salient points of the musical discourse.

The most relevant researches (Clarke & Krumhansl, 1990; Deliège, 1987, 1989; Deliège & El Ahmadi, 1990; Imberty, 1991, 1993; Lerdahl, 1989a, 1989b) insist on repetition and recursion as key elements in the organisational search. We chose this work precisely because it does not fit within the so frequent characteristics of repetition and recursion. Itinerant is a lineal work, and this research suggests that in such works we may find saliences and that experienced listeners organise their discourse through them. Thus, we confirm the salience theory with a nontonal work which does not fit within the most usual characteristics of repetition and recursion in the perceptive organisation.

## References

[bibr1-2041669517705387] Addessi A. R. Caterina R. (2005) Analysis and perception in post-tonal music: An example from Kurtág’s String Quarter Op.1. Psychology of Music 33: 94–116.

[bibr2-2041669517705387] Baroni M. (2010) GTTM and post-tonal music. Musicae Scientiae Discussion Forum 5: 69–93.

[bibr3-2041669517705387] Bianchi, F. W. (1985). *The cognition of atonal pitch structures* (Doctoral thesis). Ball State University, Muncie, Indiana.

[bibr4-2041669517705387] Clarke E. F. Krumhansl C. L. (1990) Perceiving musical time. Music Perception 7: 213–252.

[bibr5-2041669517705387] Deliège I. (1987) Grouping conditions in listening to music: An approach to Lerdahl and Jackendoff’s grouping preference rules. Music Perception 4: 325–360.

[bibr6-2041669517705387] Deliège I. (1989) A perceptual approach to contemporary musical forms. Contemporary Music Review 4: 213–230.

[bibr7-2041669517705387] Deliège I. (1993) Mechanisms of cue extraction in memory for musical time. Contemporary Music Review 9: 191–205.

[bibr8-2041669517705387] Deliège I. El Ahmadi A. (1990) Mechanisms of cue extraaction in musical groupings: A study of perception on Sequenza VI for Viola Solo by Luciano Berio. Psychology of Music 18: 18–44.

[bibr9-2041669517705387] Dibben N. (1994) The cognitive reality of hierarchic structure in tonal and atonal music. Music Perception 12: 1–25.

[bibr10-2041669517705387] Dibben N. (1999) The perception of structural stability in atonal music: The influence of salience, stability, horizontal motion, pitch commonality, and dissonance. Music Perception 16: 265–294.

[bibr11-2041669517705387] Francès, R. (1958). *La perception de la musique* [The perception of music]. Paris, France: Librairie philosophique J. Vrin.

[bibr12-2041669517705387] Gomila A. (2008) La expresión emocional en la música desde el expresionismo musical [Emotional expresión in music: A view from musical expressionism]. Estudios de Psicología 29: 117–131.

[bibr13-2041669517705387] Imberty, M. (1969). *L’acquisition des structures tonales chez l’enfant* [Acquisition of tonal structures in childhood]. Paris, France: Klincksieck.

[bibr14-2041669517705387] Imberty, M. (1990). *Le scritture del tempo. Semantica psicologica della musica.* [The writing of time. Psychological semantics of music]. Milano, Italy: Ricordi.

[bibr15-2041669517705387] Imberty M. (1991) Comment l’interprete et l’auditeur organisent-ils la progression temporelle d’une oeuvre musicale? (Analyse, memorisation et interpretation) [How do the performer and the listener organise the temporal progression of a musical work? (Analysis, memorisation and interpretation)]. Psychologica Belgica 31: 173–195.

[bibr16-2041669517705387] Imberty M. (1993) How do we perceive atonal music? Suggestions for a theoretical approach. Contemporary Music Review 9: 325–337.

[bibr17-2041669517705387] Imberty, M. (2005). *La musique creuse le temps* [Music delves into time]. Paris, France: L’Harmattan.

[bibr18-2041669517705387] Krumhansl C. L. (1992) Internal representations for music perception and performance. In: Jones M. R. Holleran S. (eds) Cognitive bases of musical communication, Washington, DC: American Psychological Association, pp. 197–211.

[bibr19-2041669517705387] Krumhansl C. L. (1996) A perceptual analysis of Mozart’s piano sonata K. 282: Segmentation, tension, and musical ideas. Music Perception 13: 401–432.

[bibr20-2041669517705387] Laucirica A. Almoguera A. Eguilaz M. J. Ordoñana J. A. (2012) El gusto por la música contemporánea en estudiantes de grado superior de conservatorio de música [Enjoyment of contemporary music in higher degree students of music conservatory]. LEEME 30: 1–20.

[bibr21-2041669517705387] Lerdahl F. (1989a) Contraintes cognitives sur les systèmes compositionnels [Cognitive constraints on compositional systems]. Actes du Symposium “Conposition et Perception Musicales”. University of Geneva, March 1987. Contrechamps 10: 25–57.

[bibr22-2041669517705387] Lerdahl, F. (1989b). Structure de prolongation dans l’atonalité [Prolongation structure in atonality]. In S. McAdams & I. Deliège (Eds.), *La musique et les sciencies cognitives* [Music and cognitive sciences] (pp. 103–135). Brussels, Belgium. Pierre Mardaga.

[bibr23-2041669517705387] Mateos-Moreno, D. (2014). Latent dimensions of attitudes towards contemporary music: A structural model. *Psychology of Music*. Advance online publication. doi:10.1177/0305735613513486.

[bibr24-2041669517705387] Ordoñana J. A. Laucirica A. (2010) Lerdahl and Jackendoff’s grouping structure rules in the performance of a Hindemith sonata. Spanish Journal of Psychology 13: 101–111. 20480681 10.1017/s113874160000370x

[bibr25-2041669517705387] Rahn J. (1980) Basic atonal theory, New York, NY: Longman.

[bibr26-2041669517705387] Reybrouck M. M. (2006) Musical creativity between symbolic modelling and perceptual constraints. The role of adaptive behaviour and epistemic autonomy. In: Deliège I. Wiggins G. A. (eds) Musical creativity. Multidisciplinary research in theory and practice, New York, NY: Psychology Press, pp. 42–59.

[bibr27-2041669517705387] Robinson, E. A. (2011). *Voice, itinerant, and air: A performance and analytical guide to the solo flute works of Toru Takemitsu* (Unpublished doctoral dissertation). Ball State University, Muncie, Indiana.

[bibr28-2041669517705387] Schulze K. Dowling W. J. Tillmann B. (2012) Working memory for tonal and atonal sequences during a forward and a backward recognition task. Music Perception 29: 255–267.

[bibr29-2041669517705387] Silver, B., & Kraft, N. (Producers). (2003). *Itinerant for Solo Flute* [Recorded by Robert Aitken]. On *Toru Takemitsu* [CD]. Toronto: HNH International Ltd. Naxos DDD 8.555859.

[bibr30-2041669517705387] Smith J. D. Witt J. N. (1989) Spun steel and stardust: The rejection of contemporary compositions. Music Perception 7: 169–186.

[bibr31-2041669517705387] Tillmann B. Bharucha J. J. Bigand E. (2000) Implicit learning of tonality: A self-organizing approach. Psychological Review 107: 885–913. 11089410 10.1037/0033-295x.107.4.885

[bibr32-2041669517705387] Wong P. C. M. Roy A. K. Margulis E. H. (2009) Bimusicalism: The implicit dual enculturation of cognitive and affective systems. Music Perception 27: 81–88. 20657798 10.1525/mp.2009.27.2.81 PMC2907111

